# Long-Term Crestal Bone Stability Following Immediate Implant Placement in the Esthetic Zone: A Clinical Case Series Using Ritter RI-Quadro Spiral Implants

**DOI:** 10.7759/cureus.117073

**Published:** 2026-09-28

**Authors:** Manuel de la Rosa-Garza, Gregori M Kurtzman

**Affiliations:** 1 Periodontology, Private Practice, Nuevo León, MEX; 2 Dentistry and Implantology, Private Practice, Silver Spring, USA

**Keywords:** crestal bone, dental implants, esthetic zone, immediate implant placement, long-term outcomes, marginal bone

## Abstract

Long-term implant success depends on more than implant survival. Maintenance of crestal bone and the surrounding soft tissue architecture becomes increasingly important as restorations remain in function for many years, particularly in the esthetic zone. This case series presents three patients treated with five Ritter RI-Quadro Spiral implants (Ritter Implants, San Antonio, Texas) in the anterior maxilla and followed for approximately 8-10 years. The clinical situations included immediate implant placement into extraction sites, facial osseous defects, guided bone regeneration, connective tissue grafting, platelet-rich fibrin, and partial extraction therapy. Insertion torque ranged from 35 to 45 N cm. All five implants remained functional during the observation period, and serial radiographs showed stable peri-implant crestal bone without progressive marginal bone loss, peri-implant radiolucency, or implant instability. The peri-implant soft tissues also remained clinically stable, with no biologic or mechanical complications requiring implant removal or replacement. Because the original treatment was not performed as a prospective study, standardized calibrated radiographs were not available, and bone-level changes could only be assessed qualitatively. Within those limitations, these cases demonstrate favorable long-term hard- and soft-tissue stability around the implants when used as part of a prosthetically driven approach that included appropriate regenerative therapy and maintenance. Larger prospective studies with standardized quantitative measurements are needed to determine how much of the observed stability can be attributed to implant design versus other surgical, restorative, and patient-related factors.

## Introduction

Long-term implant success is no longer judged by survival alone. An implant may remain integrated and functional while still demonstrating crestal bone remodeling, soft tissue recession, or changes that compromise the restorative result. This is especially relevant in the anterior maxilla, where relatively small changes in supporting bone can affect gingival architecture and esthetics.

Crestal bone maintenance is influenced by biologic, surgical, restorative, and biomechanical factors. Implant surface topography influences the biologic response at the bone-implant interface, with moderately rough titanium surfaces generally demonstrating a favorable response during osseointegration [1]. Primary stability is influenced by implant design, surgical technique, and local bone quality [2]. Implant macrogeometry can affect initial mechanical engagement and stability [3], while implant geometry and surface characteristics may also influence insertion torque and primary stability [4]. The importance of adequate primary stability for successful osseointegration has been well established [5]. The relationship between implant surface characteristics and osseointegration continues to be supported in contemporary review literature [6].

Restorative and implant-abutment variables may also influence peri-implant bone stability. Conical implant-abutment connections have demonstrated favorable mechanical and biologic characteristics [7], while platform switching has been associated with reduced marginal bone loss compared with platform-matched connections [8]. Abutment height may influence marginal bone remodeling [9], and the characteristics of the transmucosal implant surface can affect soft-tissue attachment and marginal bone response [10]. Prosthetic design has also been associated with peri-implant bone-level changes [11], including the potential influence of crown dimensions on marginal bone stability [12].

Implant-neck design represents another potential factor. Long-term clinical evidence comparing implants with and without cervical microthreads has not demonstrated a significant difference in marginal bone loss or implant survival [13]. In addition, the vertical position of platform-switched conical-connection implants relative to the crestal bone may influence subsequent marginal bone remodeling [14].

## Case presentation

Ritter RI-Quadro Spiral implant

The Ritter RI-Quadro Spiral implant (Ritter Implants, San Antonio, Texas) combines a tapered body with an aggressive spiral thread design, cervical microthreads, a moderately rough sandblasted large-grit acid-etched surface, and an internal conical restorative connection (Figure 1). These manufacturer-described features are designed to increase bone engagement at placement and provide a stable restorative interface; however, the present case series did not isolate or test the independent effect of any design feature. The clinical question, however, is not simply whether these design features are theoretically favorable, but whether the surrounding bone and soft tissues remain stable after years of function.

**Figure 1:**
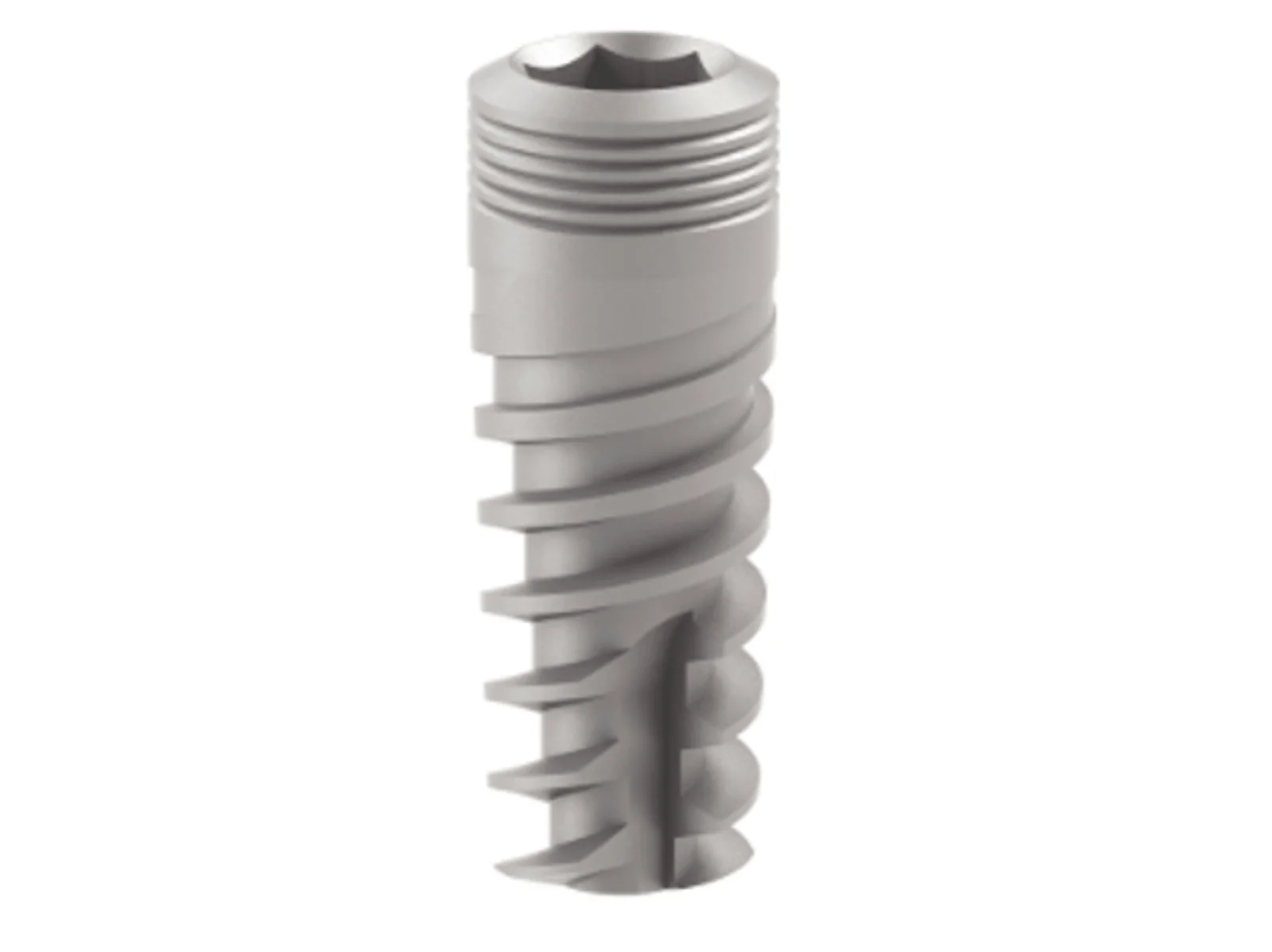
Ritter RI-Quadro Spiral implant demonstrating the tapered body and progressive spiral thread design intended to enhance bone engagement and primary stability. Image provided by Ritter Implants (San Antonio, Texas).

Long-term peer-reviewed clinical documentation specifically involving this implant remains limited. The purpose of this descriptive report is to document 8- to 10-year clinical and radiographic observations from five Ritter RI-Quadro Spiral implants placed in three patients in the anterior maxilla. It addresses the current lack of long-term, peer-reviewed clinical documentation for this implant without testing implant-specific efficacy or comparing it with another implant system. Particular attention is directed to crestal bone maintenance, peri-implant tissue stability, and continued prosthetic function.

Case presentation

Three patients were treated with a total of five Ritter RI-Quadro Spiral implants in the anterior maxilla between September 2015 and February 2016. All implant positions were planned prosthetically and placed with the aid of a surgical guide. Immediate placement was used following extraction, with hard- and soft-tissue augmentation added according to the defect and the esthetic requirements of each site. The patients reviewed the proposed treatment plans and provided written informed consent before treatment.

All implants had a tapered body, aggressive spiral thread geometry, and a moderately rough sandblasted, large-grit, acid-etched surface. Primary stability was recorded as insertion torque at placement. The clinical records and available serial radiographs were reviewed for implant survival, continued prosthetic function, peri-implant soft tissue appearance, and maintenance of supporting crestal bone.

The original treatment was performed as clinical care rather than as a prospective radiographic study. Standardized calibrated baseline and follow-up radiographs were therefore not available.

For that reason, crestal bone changes are described qualitatively rather than reported as millimeter measurements. Periapical radiographs were used for serial follow-up because they provide high-resolution assessment with lower radiation exposure; cone-beam computed tomography (CBCT) was reserved for three-dimensional diagnostic questions and was not obtained solely to measure routine follow-up crestal bone levels.

At the final follow-up evaluation in all three cases, peri-implant probing depths were 2-3 mm. Bleeding on probing and percussion findings were within normal limits for clinically healthy implants. These clinical observations were considered together with the serial radiographic findings.

Case series

Case 1

The patient was a 47-year-old man who was a nonsmoker and systemically healthy, with a generally healthy periodontal status except at tooth #9, where a failing endodontic-periodontal lesion was present. Tooth #7 had a nonrestorable fracture. Tooth #8 had been missing before presentation, and the patient had a fixed bridge from #7 to #9 replacing #8. The bridge was fractured, and the remaining tooth structure on #7 and #9 was insufficient to support a new tooth-supported bridge (Figure 2). After atraumatic extraction under intravenous sedation, the osteotomies were prepared using a surgical guide, and two implants were placed immediately.

**Figure 2:**
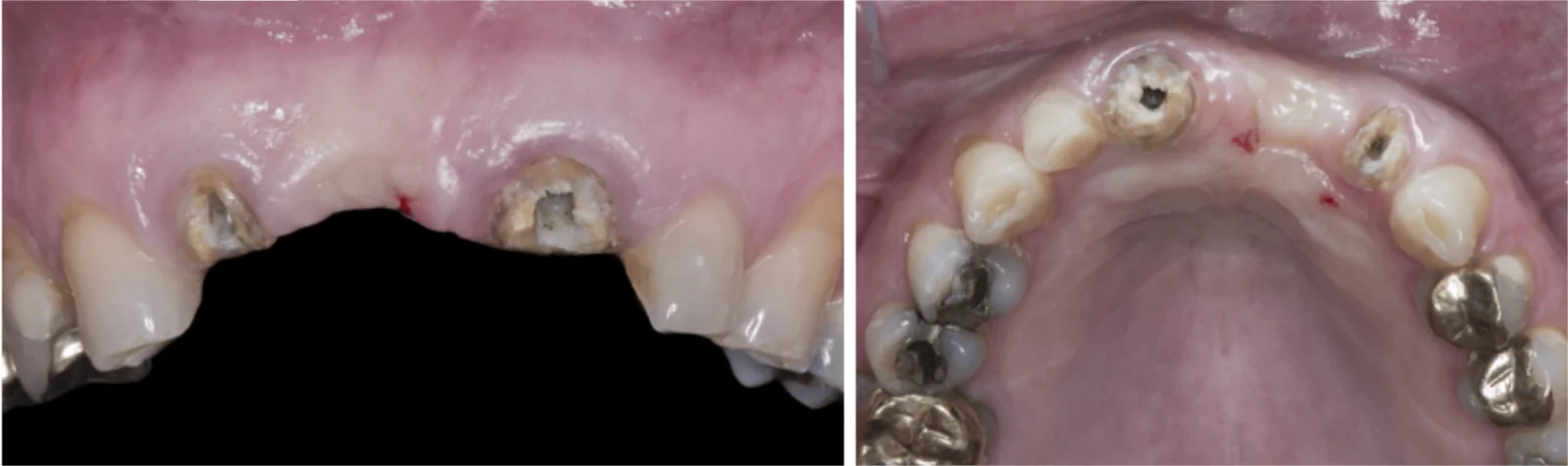
Clinical appearance after removal of the failed anterior fixed prosthesis, demonstrating the previously endodontically treated teeth before extraction.

The treatment plan was therefore to replace teeth #7 and #9 with implants that would support a fixed prosthesis replacing the missing #8. The available records did not provide a standardized periodontal/peri-implant maintenance interval for this retrospective report.

A 3.75-mm-diameter implant was placed at #7 and a 4.2-mm-diameter implant at #9. Both implants achieved approximately 40 N cm of insertion torque. The facial defect at #9 was treated with particulate bovine xenograft (Bio-Oss; Geistlich Pharma AG, Wolhusen, Switzerland) and a Bio-Gide resorbable collagen membrane (Geistlich Pharma AG). A connective tissue graft was harvested from the posterior palate and was also placed to increase soft tissue thickness and support the peri-implant tissue architecture.

At approximately 10 years, both implants remained functional. The follow-up periapical radiograph demonstrated generally maintained crestal bone around the implants (Figure 3). Clinically, the peri-implant tissues remained stable, with slight recession at the lateral incisor site but no visible inflammation (Figure 4). At final follow-up, probing depths were 2-3 mm at the implant sites, bleeding on probing was within normal limits for clinically healthy implants, and percussion testing revealed no abnormal findings.

**Figure 3:**
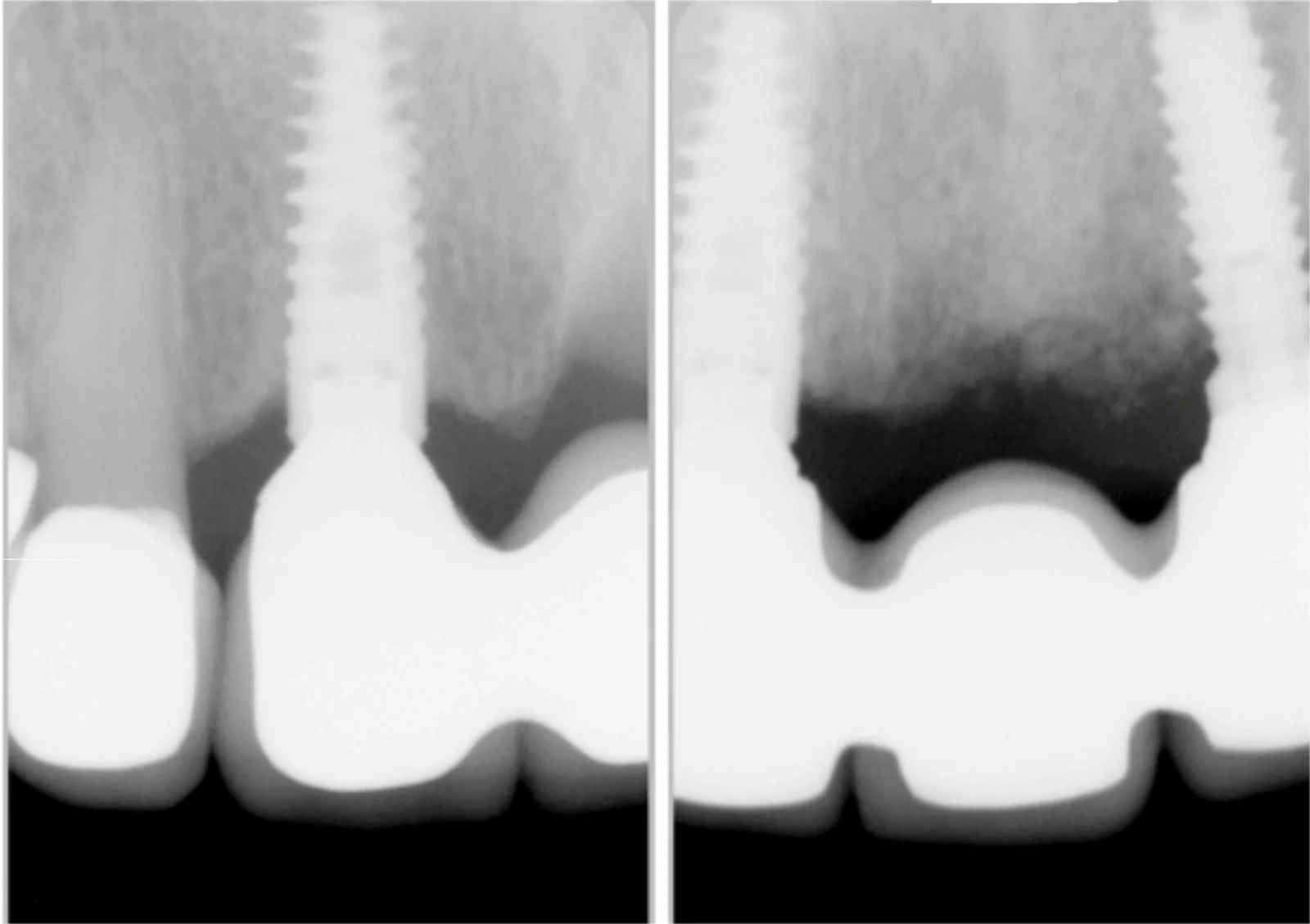
Periapical radiograph at approximately 10 years demonstrating stable crestal bone around the implants.

**Figure 4:**
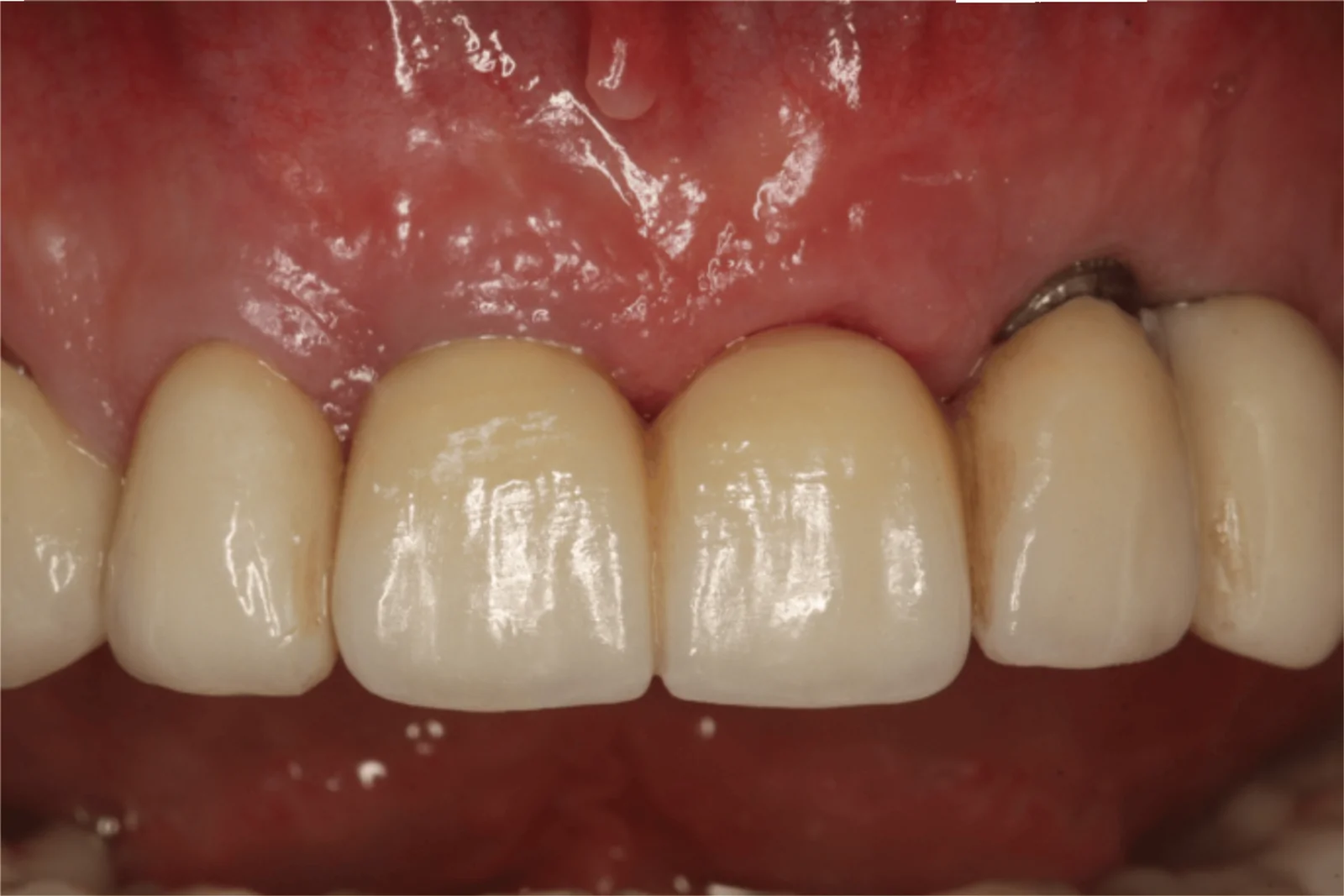
Clinical appearance at approximately 10 years showing stable marginal tissue around the central incisor implant and slight recession at the lateral incisor implant without visible inflammation.

A resin-bonded Maryland-type provisional restoration was used during healing. The definitive restoration was a screw-retained implant-supported fixed bridge. The exact interval to definitive restoration was not documented in the information available for this retrospective report. 

Case 2

The second patient was a 34-year-old woman who was a nonsmoker and systemically healthy with a healthy periodontal status. Tooth #8 was a failing endodontically treated maxillary central incisor with a relatively short root. The preoperative periapical radiograph demonstrated associated periapical pathology (Figure 5), and cone-beam computed tomography showed loss of the facial plate (Figure 6). Because the extent of the endodontic lesion, limited remaining tooth structure expected after retreatment, and root dimensions resulted in a poor restorative prognosis, atraumatic extraction and immediate implant placement were selected. A surgical guide was used for osteotomy preparation, and a 4.2-mm Ritter RI-Quadro Spiral implant was placed immediately without flap elevation. The available records did not provide a standardized periodontal/peri-implant maintenance interval for this retrospective report.

**Figure 5:**
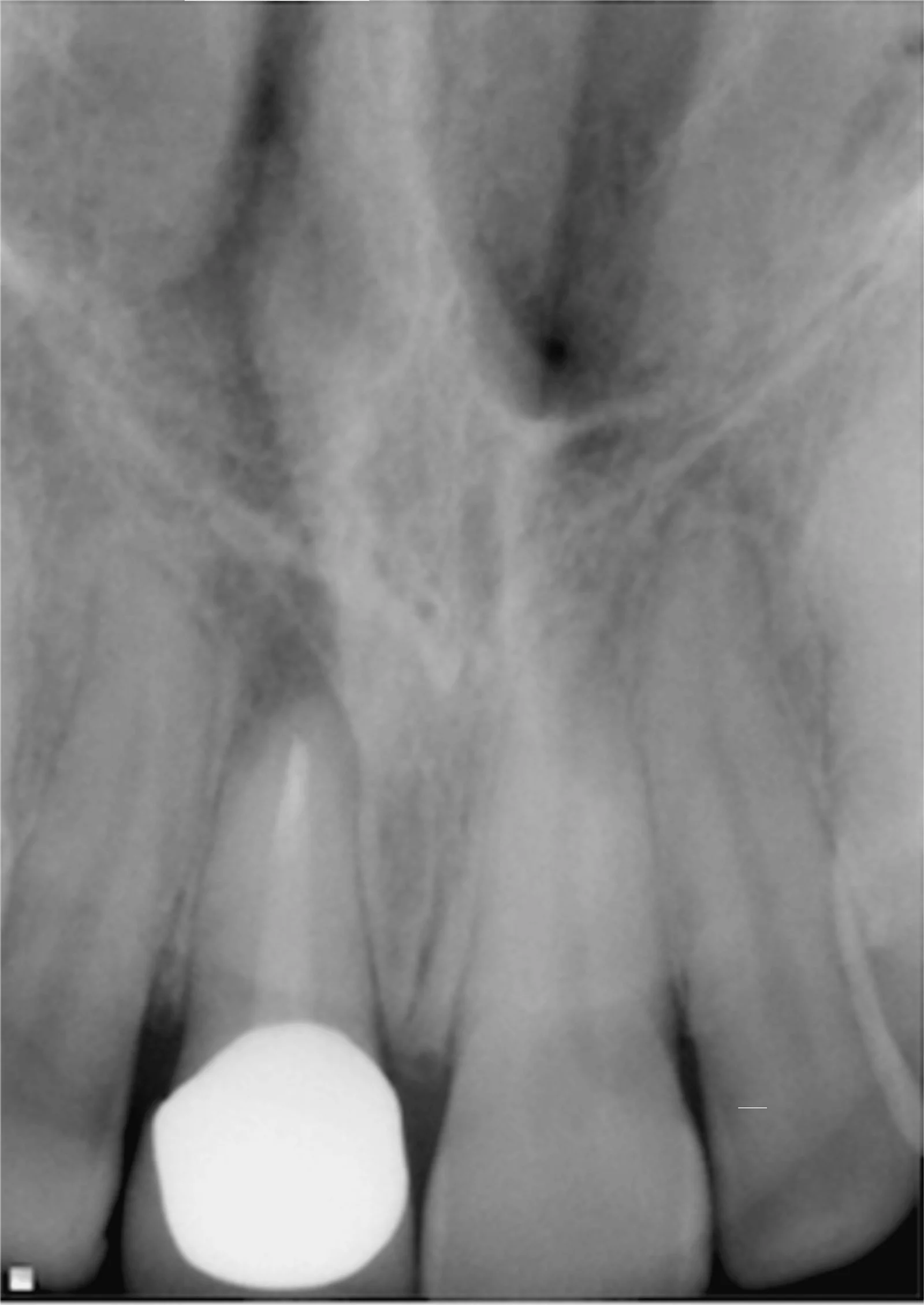
Preoperative periapical radiograph demonstrating the failing endodontically treated maxillary central incisor with associated periapical pathology.

**Figure 6:**
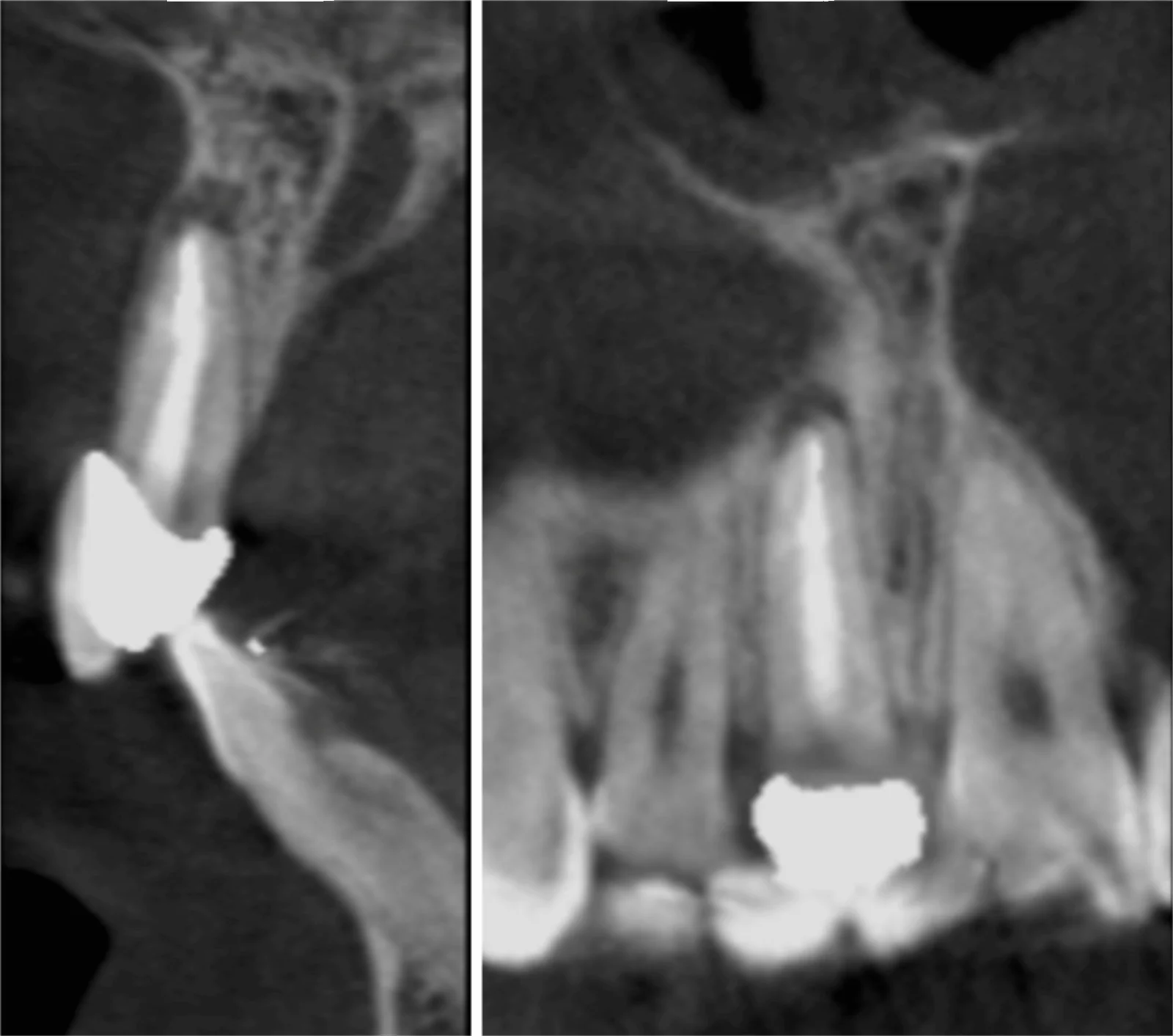
Preoperative cone-beam computed tomography demonstrating absence of the facial plate at the failing maxillary central incisor.

The implant was positioned palatally, leaving an approximately 2-mm facial gap that could be grafted before closure (Figure 7). The implant achieved approximately 45 N cm of insertion torque. Regeneration included particulate bovine xenograft (Bio-Oss; Geistlich Pharma AG, Wolhusen, Switzerland) combined with autogenous bone and platelet-rich fibrin. A connective tissue graft from the palate was added to reinforce the facial soft tissue volume.

**Figure 7:**
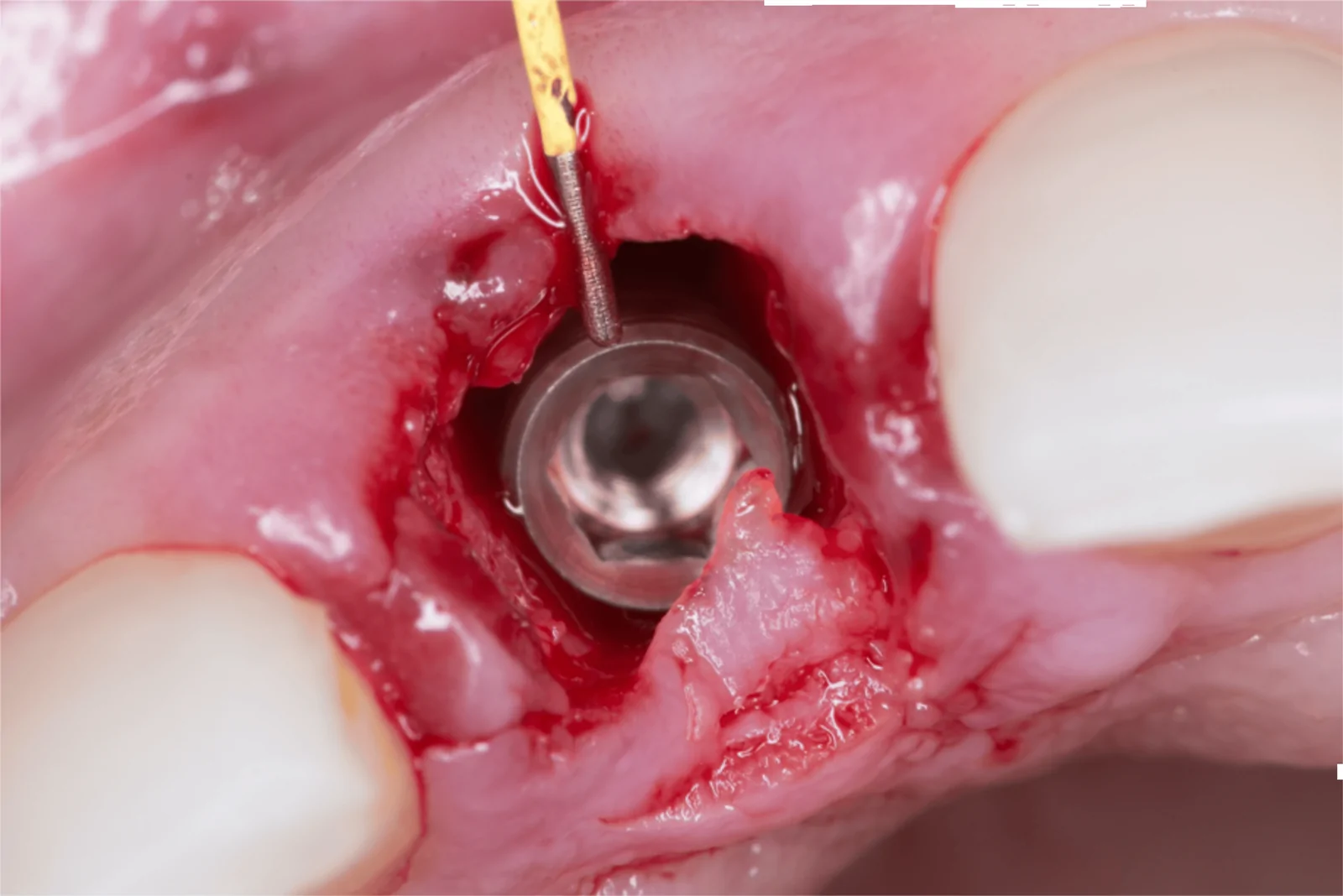
Immediate implant placement in a palatal position after extraction, leaving a facial gap for grafting before flap closure.

The implant position at placement is shown in Figure 8, and a radiograph obtained at 15 weeks documents healing (Figure 9). A resin-bonded Maryland-type provisional restoration was placed at surgery and left out of functional loading during healing. After five months, the implant was restored with a screw-retained definitive implant crown.

**Figure 8:**
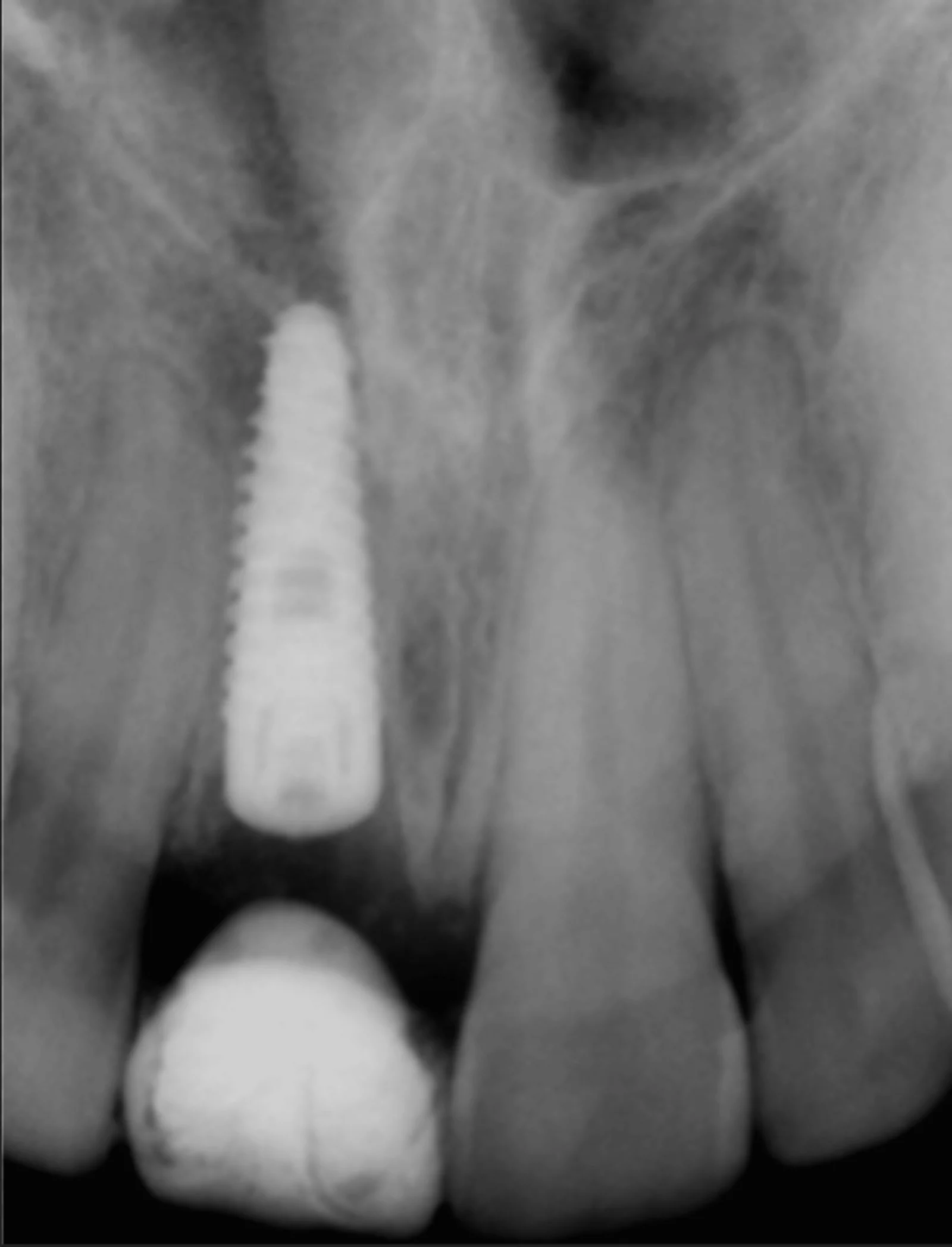
Periapical radiograph obtained at implant placement.

**Figure 9:**
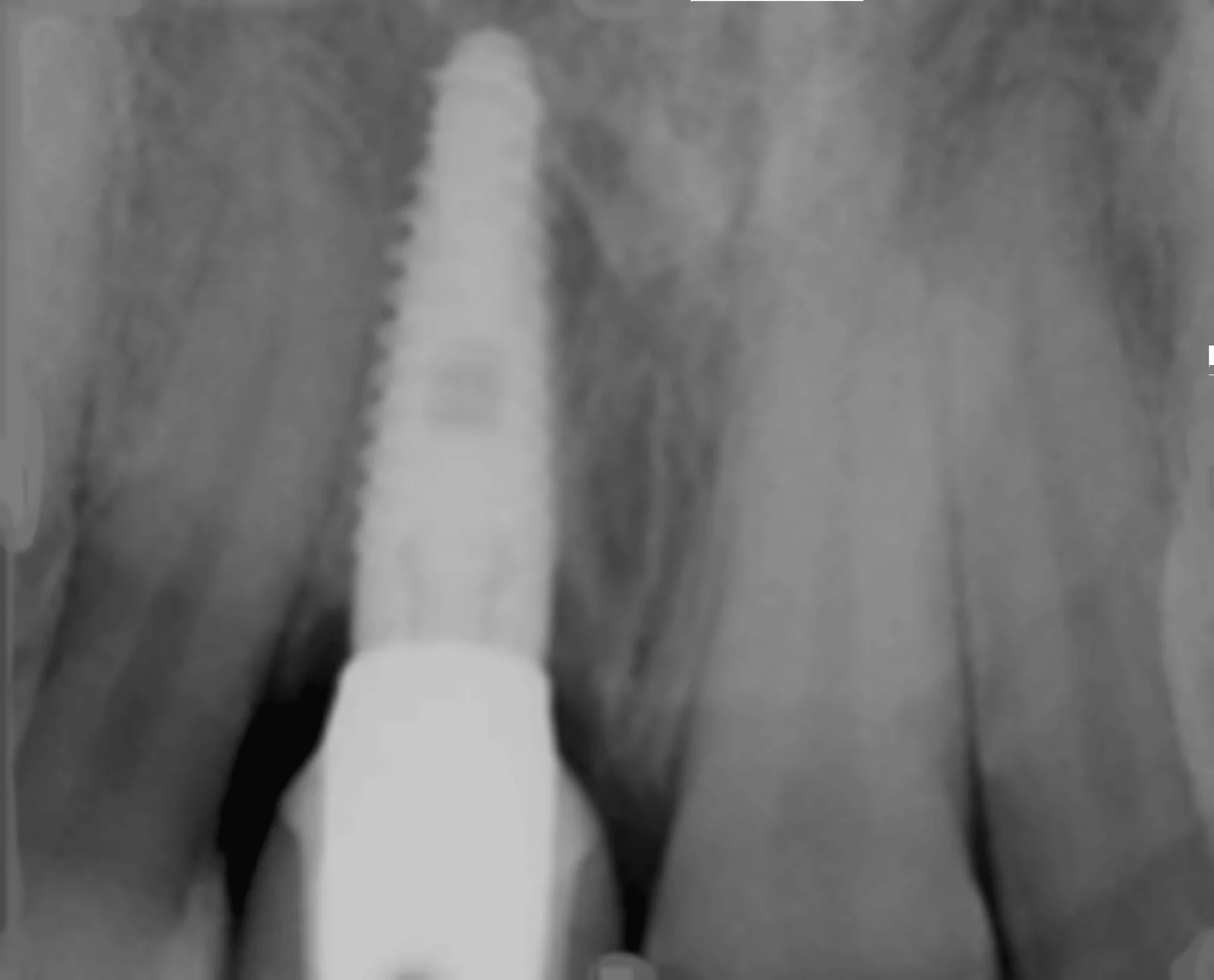
Periapical radiograph obtained 15 weeks after implant placement.

At final follow-up, probing depths were 2-3 mm at the implant site, bleeding on probing was within normal limits for a clinically healthy implant, and percussion testing revealed no abnormal findings.

At approximately 10 years, the restoration remained in function, and the marginal soft tissues appeared stable without visible inflammation (Figure 10). The long-term periapical radiograph showed preservation of crestal bone support without a generalized pattern of progressive radiographic bone loss or peri-implant radiolucency (Figure 11).

**Figure 10:**
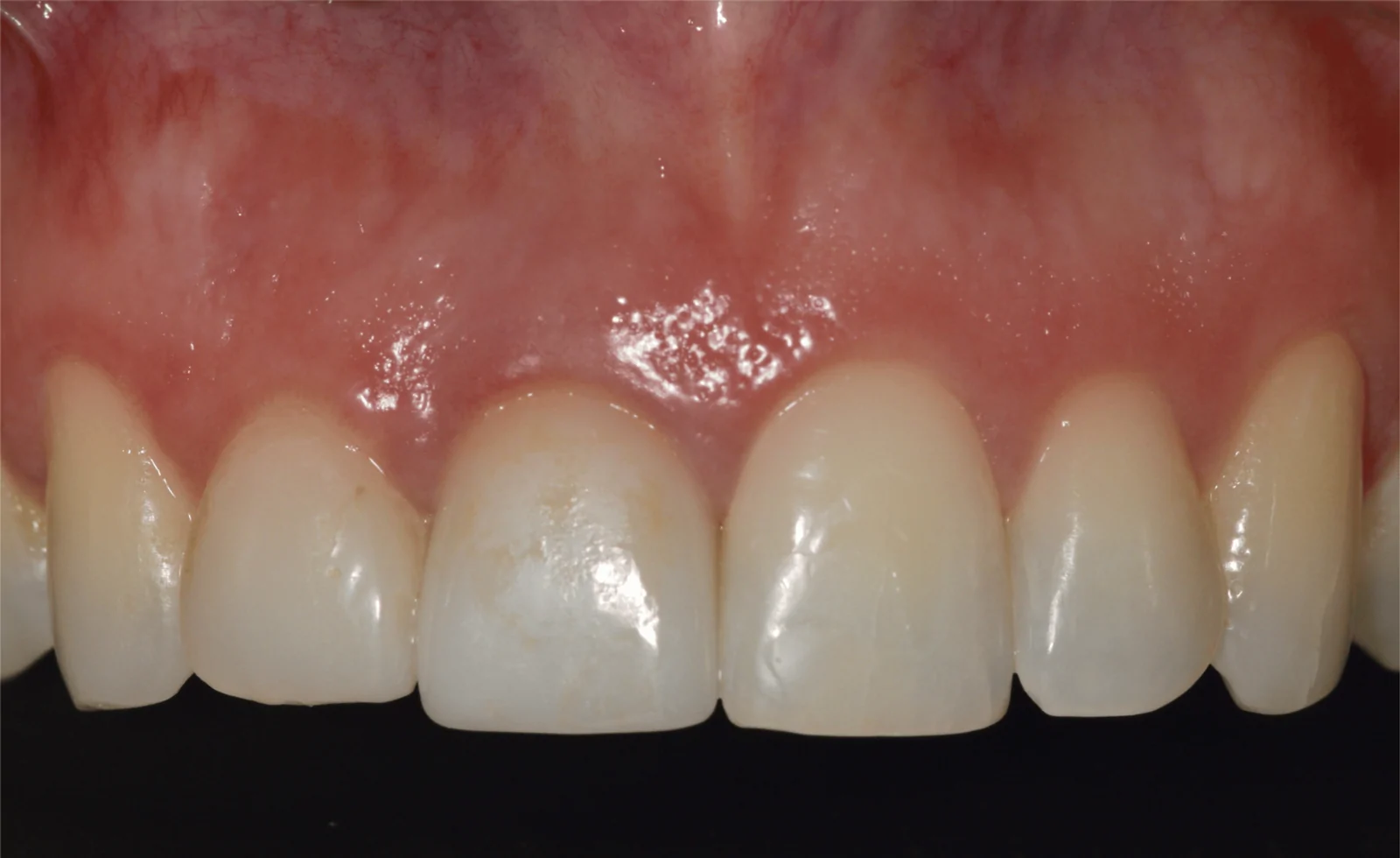
Clinical appearance at approximately 10 years showing stable marginal gingiva without visible inflammation.

**Figure 11:**
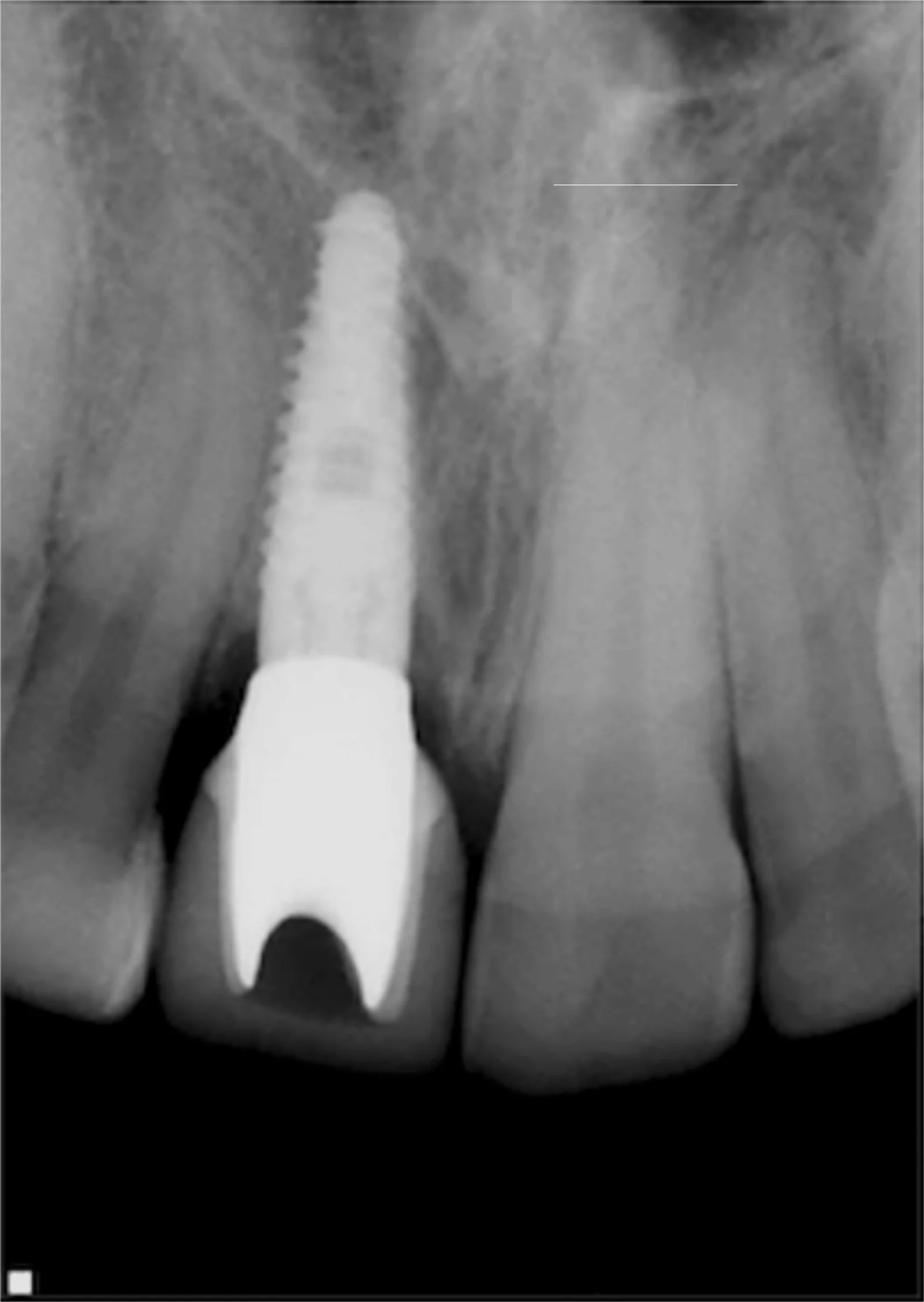
Periapical radiograph at approximately 10 years demonstrating maintenance of crestal bone around the implant.

Case 3

The third patient was a 28-year-old woman who was a nonsmoker and systemically healthy with a healthy periodontal status. Tooth #8 had been lost approximately three years earlier following infection without endodontic treatment and was an edentulous pontic site at presentation. Tooth #9 had undergone endodontic treatment approximately two years earlier, but pain developed about one year later. On reevaluation, the prognosis for additional endodontic treatment of #9 was considered poor. The treatment plan therefore included atraumatic extraction and immediate implant placement at #9 together with implant placement at the previously edentulous #8 site (Figures 12, 13). The available records did not provide a standardized periodontal/peri-implant maintenance interval for this retrospective report.

**Figure 12:**
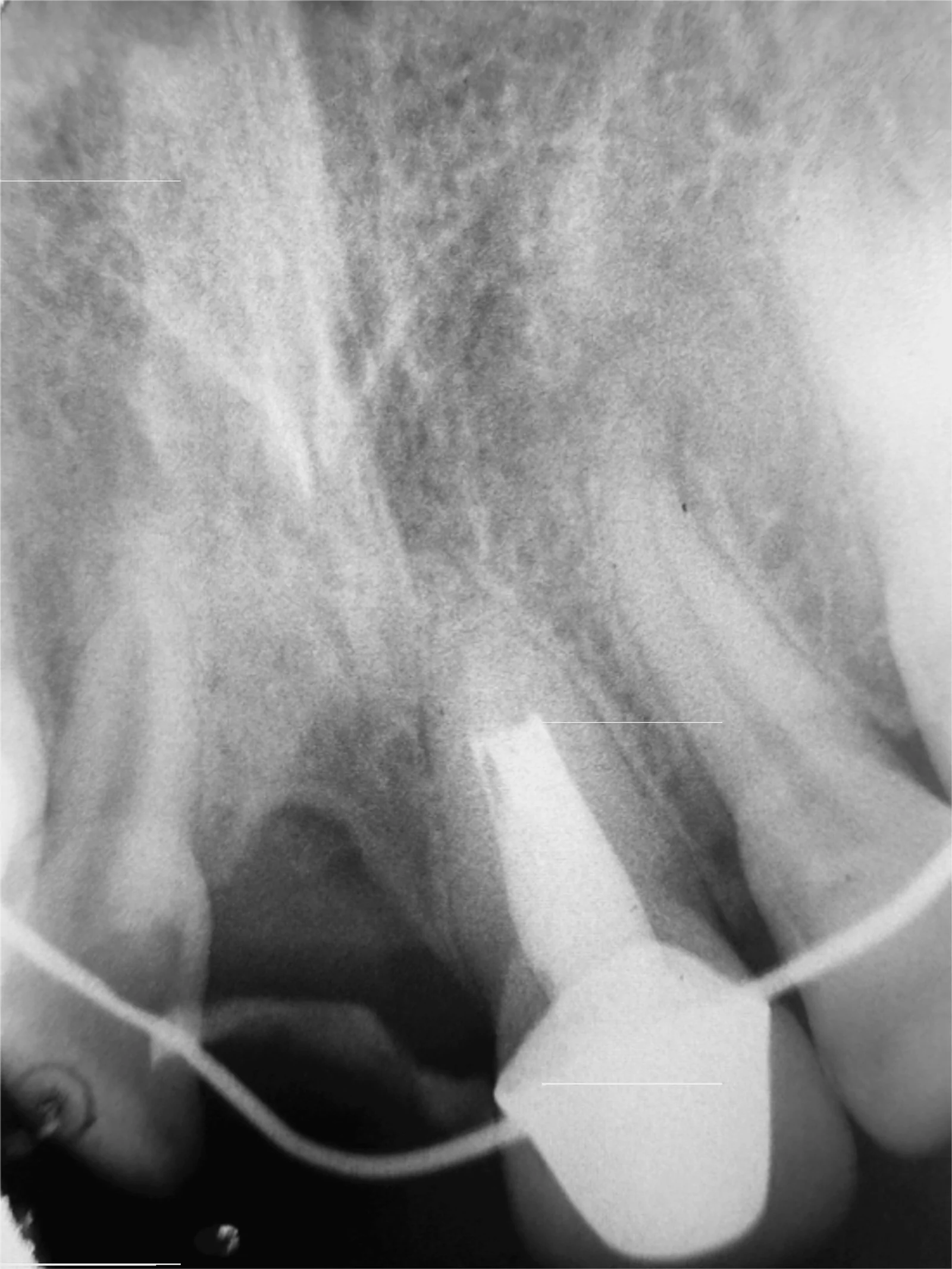
Preoperative periapical radiograph demonstrating the failing endodontically treated maxillary central incisor before extraction and implant placement.

**Figure 13:**
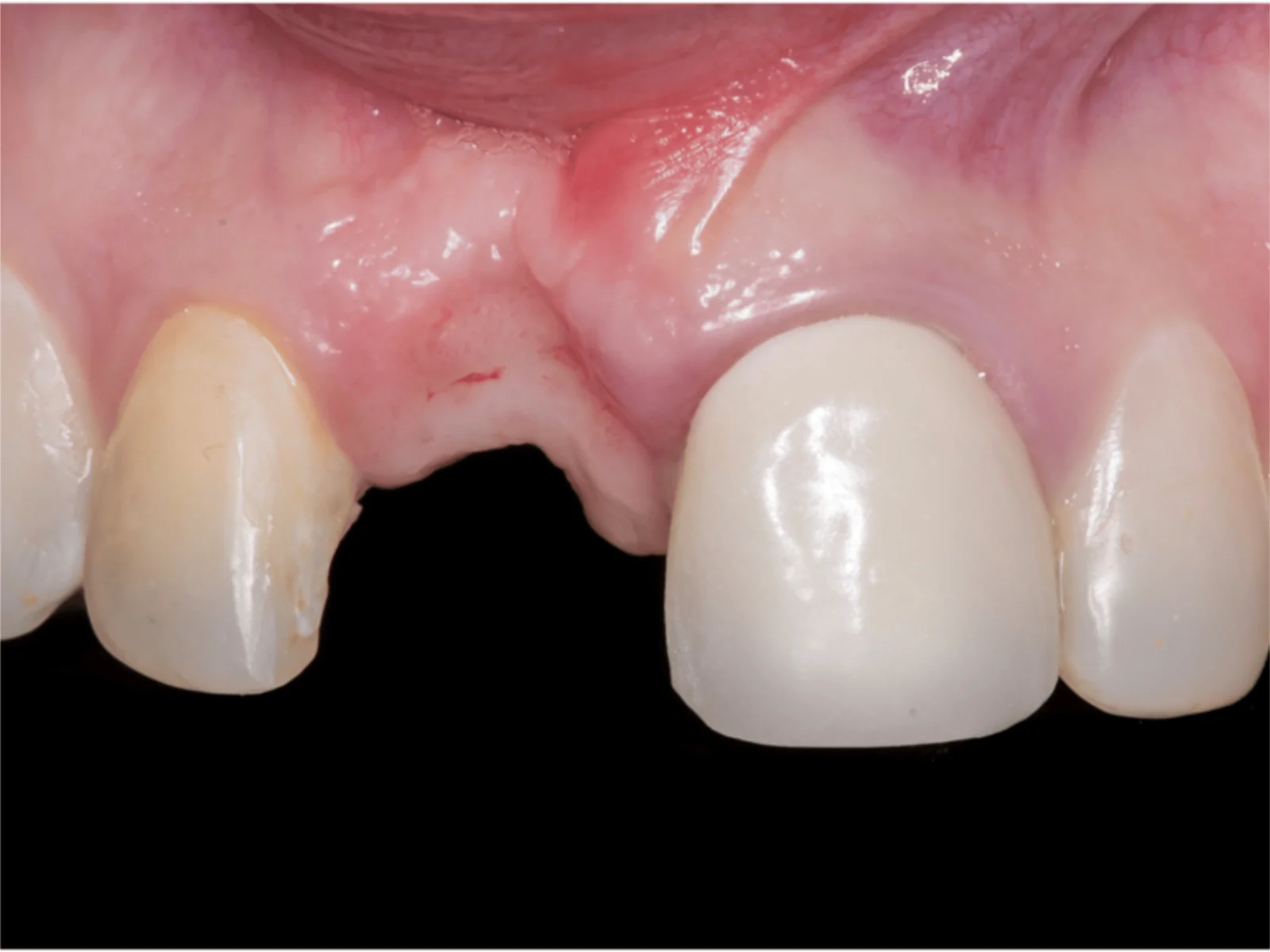
Pre-treatment clinical appearance of the maxillary anterior site.

After prosthetically guided treatment planning, immediate implant placement at #9 was combined with partial extraction therapy using a socket-shield approach to help preserve the facial periodontal attachment apparatus; a second implant was placed at the healed #8 site.

Two 4.2-mm Ritter RI-Quadro Spiral implants were placed with insertion torque values of approximately 35 N cm. Particulate bovine xenograft (Bio-Oss; Geistlich Pharma AG, Wolhusen, Switzerland) was combined with autogenous bone and covered with a Bio-Gide resorbable collagen membrane (Geistlich Pharma AG). Connective tissue grafts harvested from the posterior palate were placed on the facial aspect of both implant sites to increase soft-tissue thickness in the esthetic zone.

Early radiographs at six weeks and three months demonstrated maintenance of the peri-implant crestal bone during healing (Figures 14, 15). A resin-bonded Maryland-type provisional restoration was used during healing.

**Figure 14:**
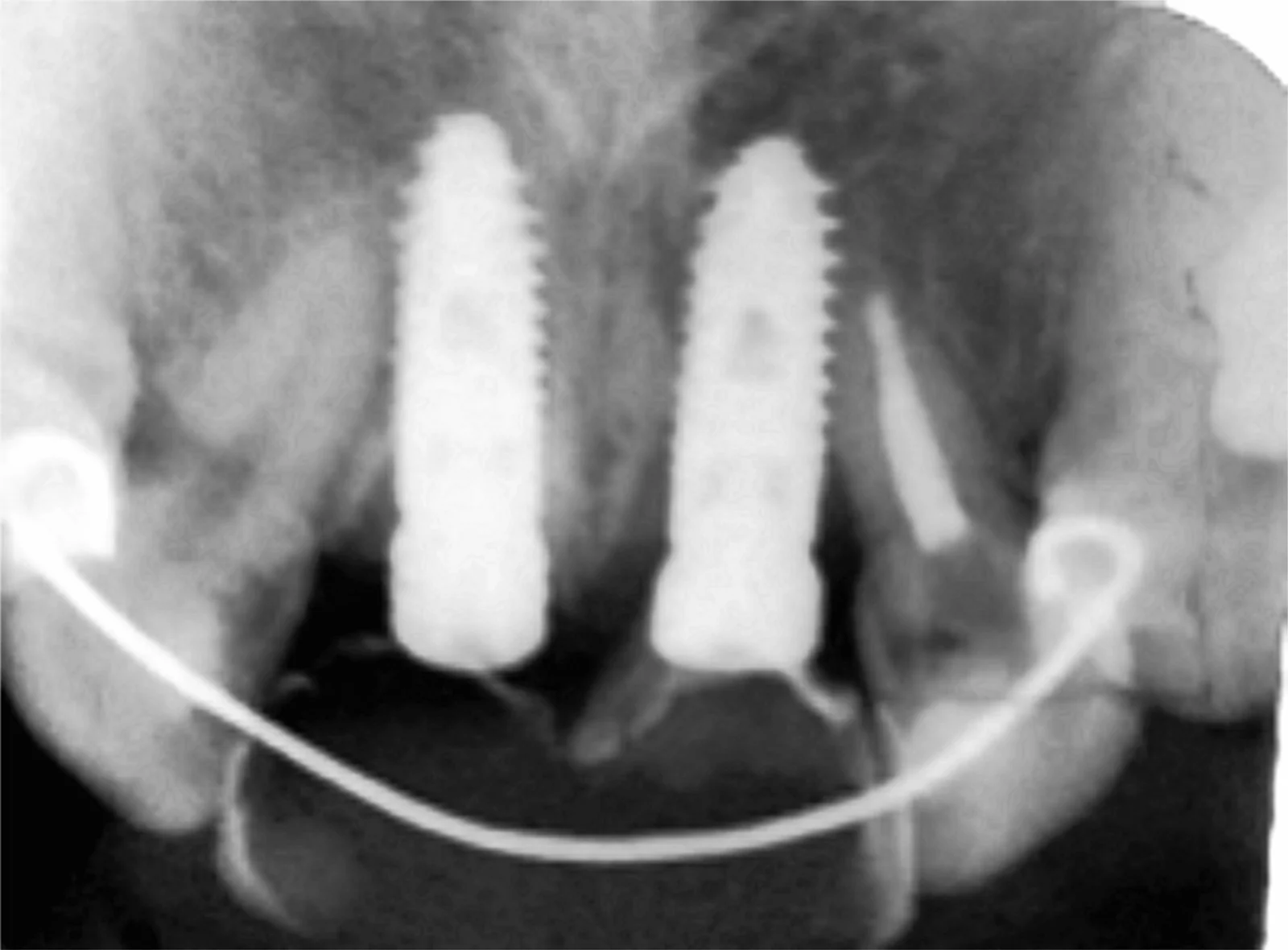
Periapical radiograph obtained six weeks after implant placement.

**Figure 15:**
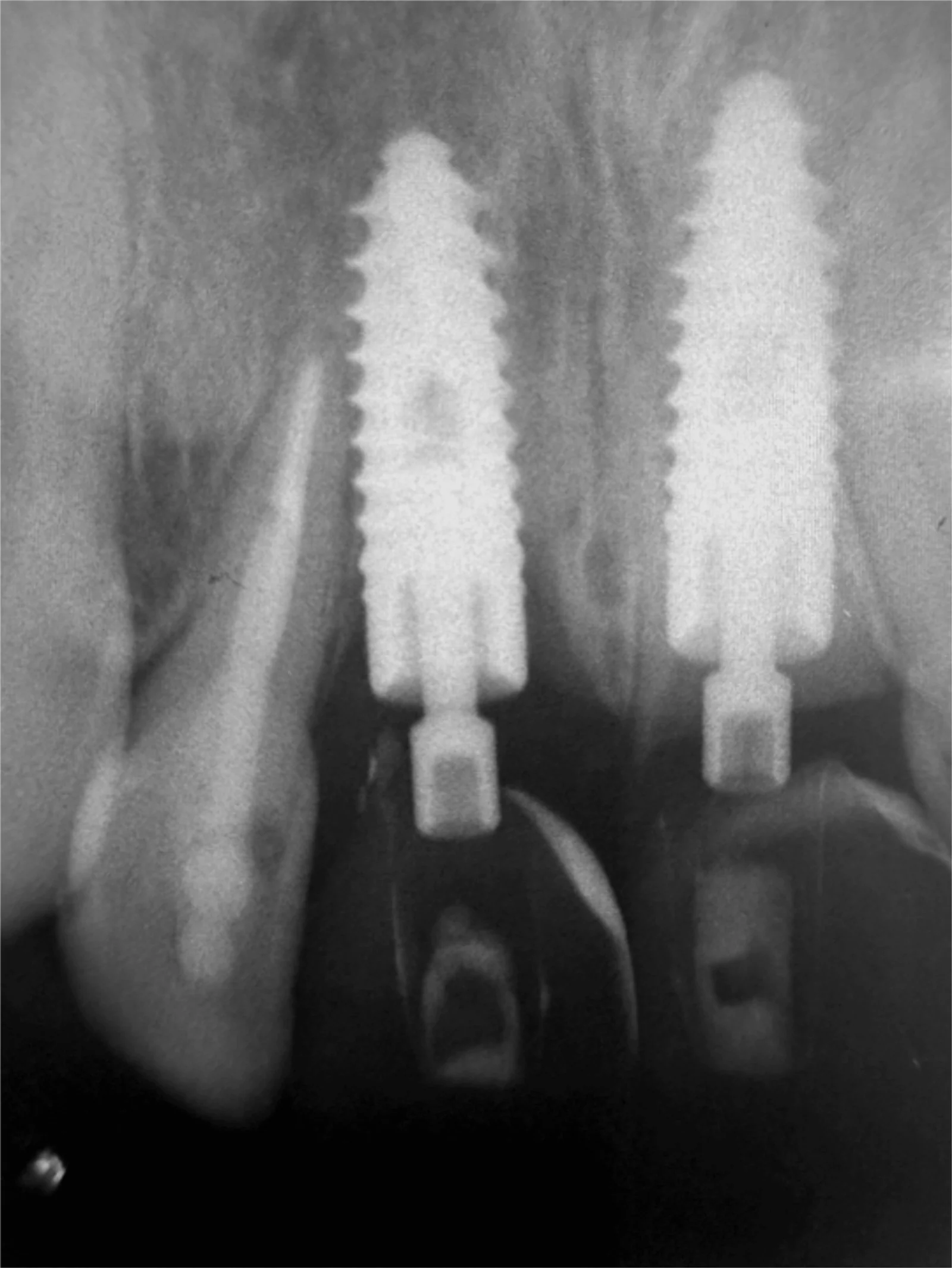
Periapical radiograph at three months demonstrating maintenance of peri-implant crestal bone during early healing.

At six months, final impressions were made and screw-retained porcelain implant crowns were placed (Figure 16). At eight years, the marginal tissues remained clinically stable without visible inflammation (Figure 17), and the periapical radiograph continued to show generally maintained crestal architecture around both implants (Figure 18).

**Figure 16:**
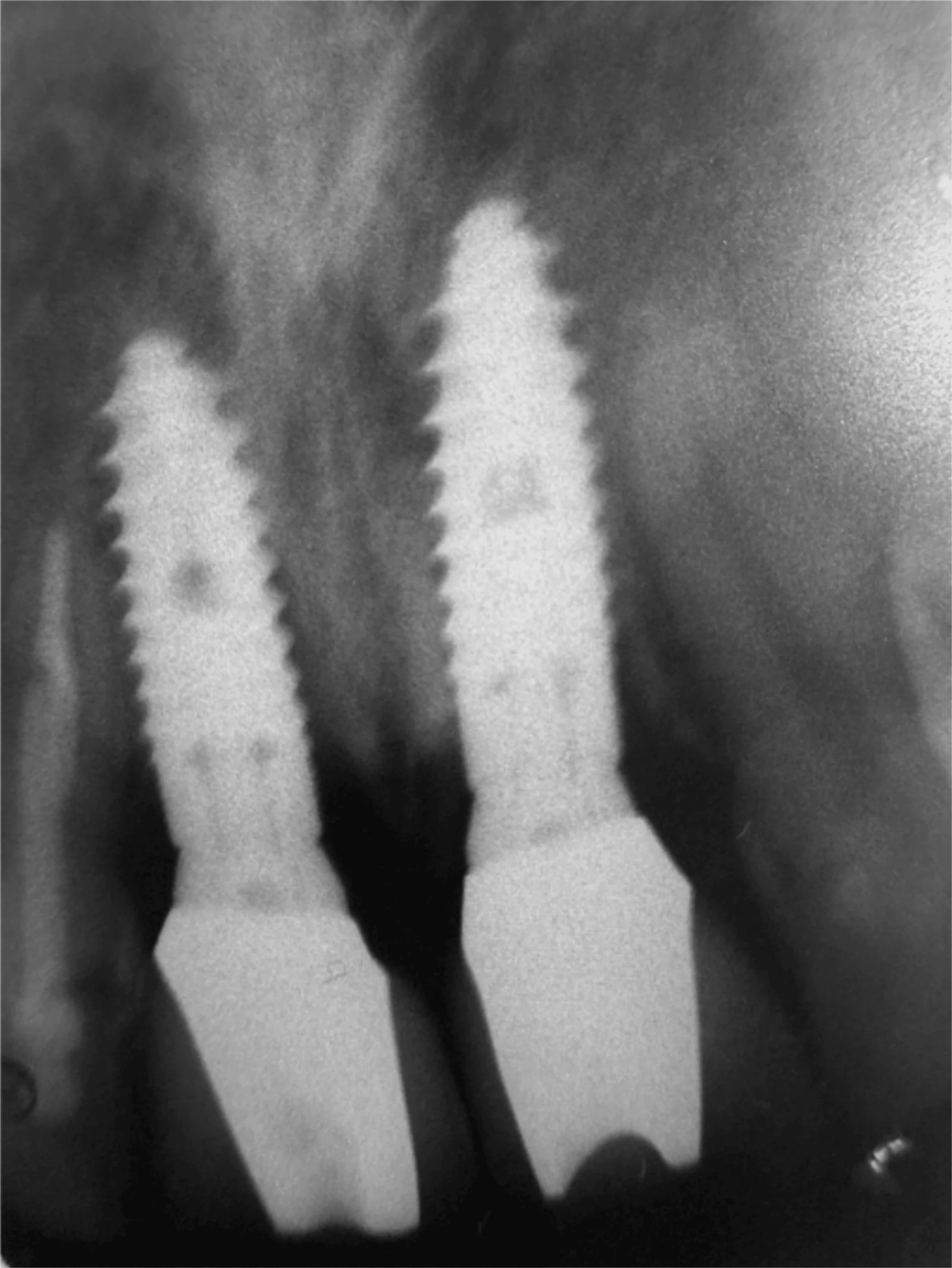
Periapical radiograph at definitive restoration placement demonstrating maintained crestal bone around both implants.

**Figure 17:**
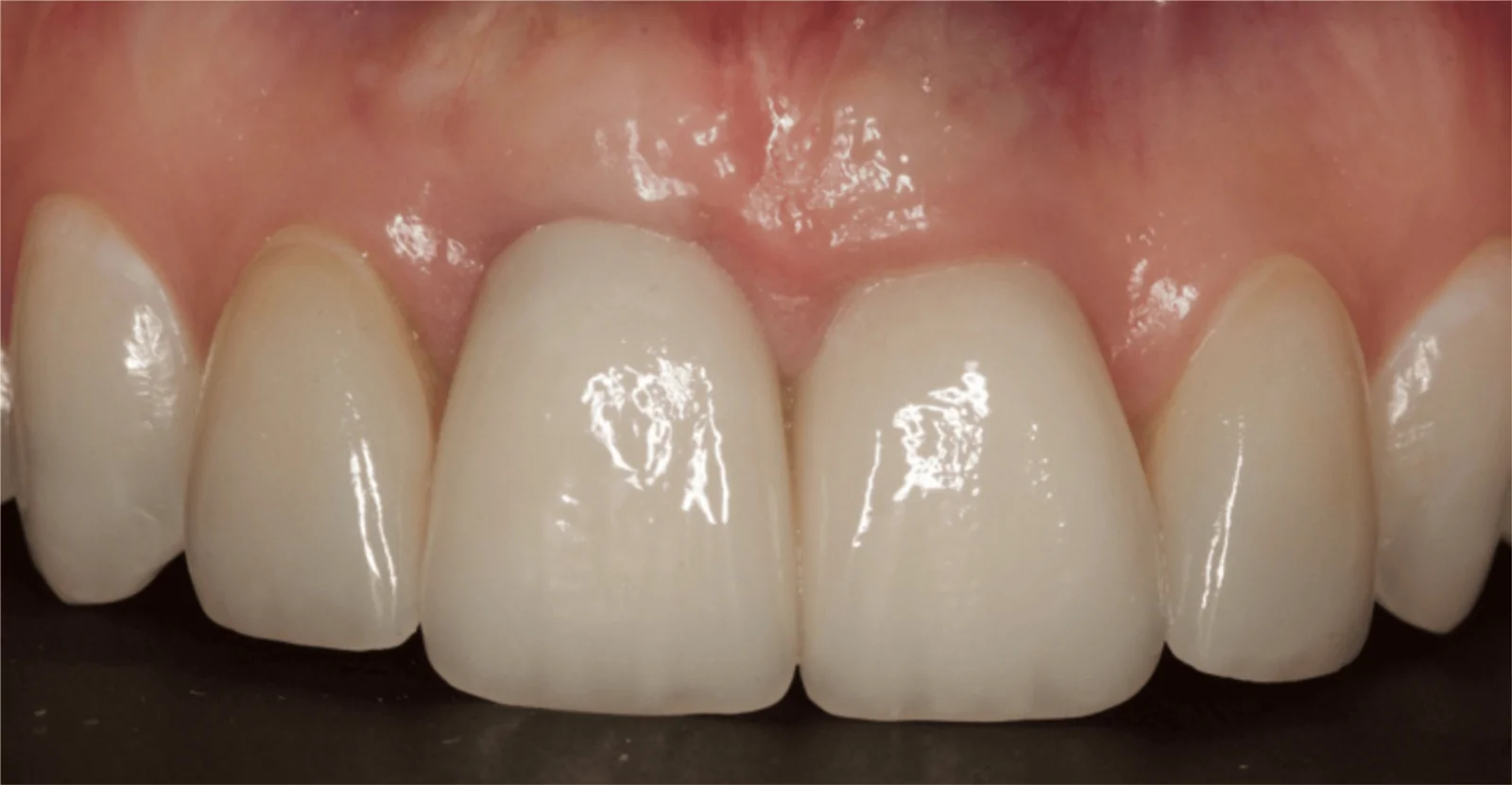
Clinical appearance at eight years demonstrating stable marginal gingiva without visible inflammation.

**Figure 18:**
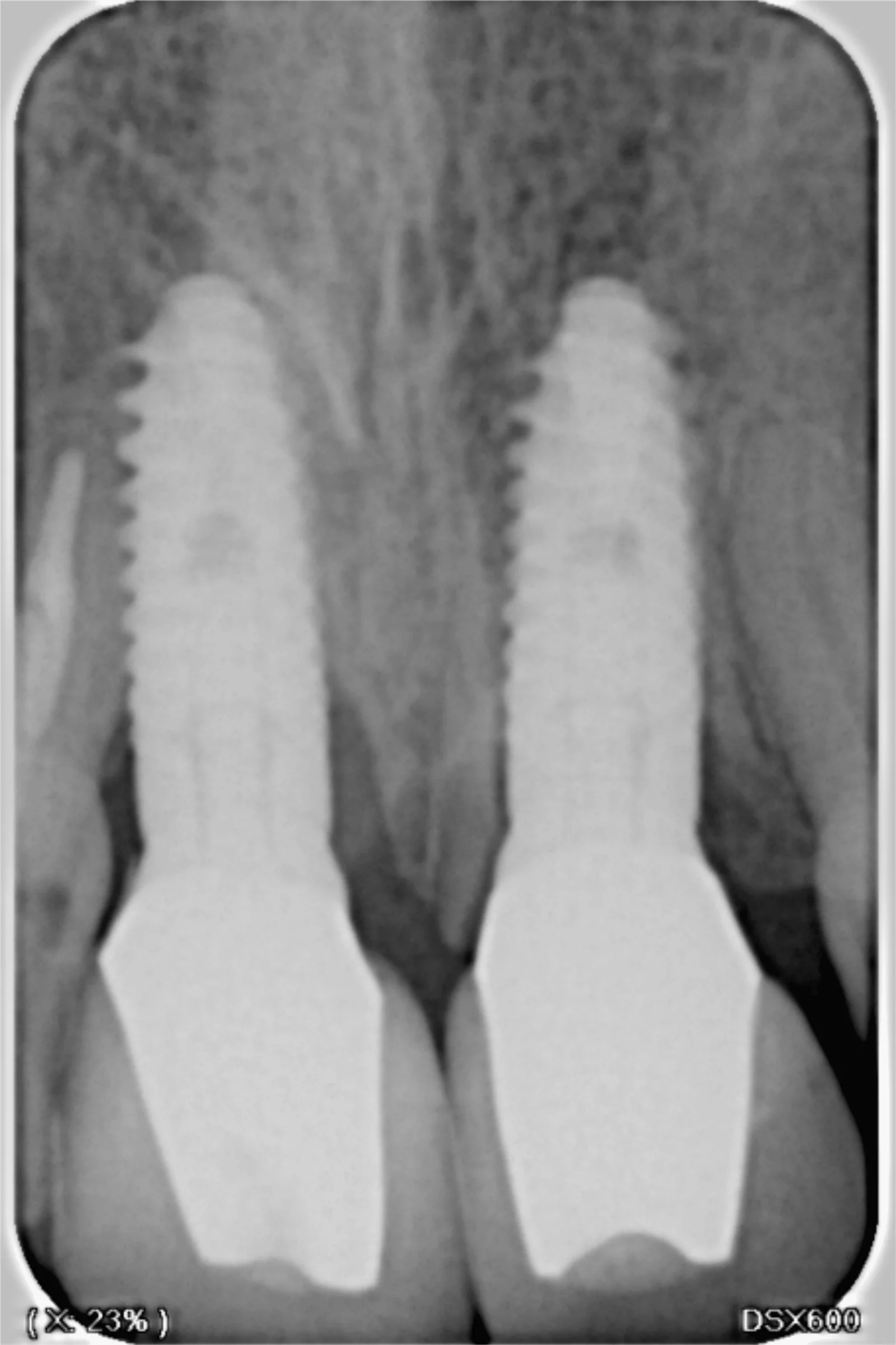
Periapical radiograph at eight years demonstrating stable crestal bone around both implants.

At implant #8, the long-term periapical radiograph shows an angular reduction of the mesial crestal bone level; the distal level appears comparatively similar. Because baseline and follow-up radiographs were not standardized or calibrated, the magnitude, timing, progression, and cause of this change cannot be determined reliably. It is therefore reported as a site-specific qualitative observation rather than attributed to implant design. At final follow-up, probing depths were 2-3 mm at both implant sites, bleeding on probing was within normal limits for clinically healthy implants, and percussion testing revealed no abnormal findings.

Case Series Outcomes

Across the three patients, five implants were followed for approximately eight to 10 years. Implant dimensions, insertion torque, regenerative procedures, and follow-up are summarized in Table 1. All five implants remained in function (5/5) during the available follow-up. This proportion is descriptive only and should not be interpreted as a generalizable survival estimate. Recorded insertion torque ranged from 35 to 45 Ncm.

**Table 1:** Clinical summary of the case series. GBR: guided bone regeneration, CT: connective tissue, PRF: platelet-rich fibrin.

Case	Implant Sites	Number of Implants	Diameter (mm)	Torque (N cm)	Regenerative Procedure	Follow-up	Implant Survival	Bone Maintenance
1	#7, #9	2	3.75, 4.2	40	GBR + Bio-Oss + Bio-Gide collagen membrane + CT graft	~10 years	Yes	Generally maintained radiographically
2	#8	1	4.2	45	Bio-Oss + autogenous bone + PRF + CT graft	~10 years	Yes	Generally maintained radiographically
3	#8, #9	2	4.2	35	Socket shield + GBR + Bio-Oss + Bio-Gide collagen membrane + CT graft	~8 years	Yes	#8 mesial angular change; other sites generally maintained

The long-term clinical findings are summarized in Table 2. With the exception of a site-specific angular mesial crestal change at implant #8 in Case 3, the available nonstandardized radiographs did not show a pattern of progressive marginal bone loss, peri-implant radiolucency, or loss of stability. No biologic or mechanical complication required implant removal or replacement. The peri-implant soft tissue architecture also remained clinically acceptable around the restorations.

**Table 2:** Long-term clinical outcomes following placement of Ritter RI-Quadro Spiral (QSI) implants (Ritter Implants, San Antonio, Texas).

Outcome Measure	Case 1	Case 2	Case 3	Overall Findings
Follow-up duration	~10 years	~10 years	~8 years	Mean ≈10 years
Number of implants	2	1	2	5
Implant survival	100%	100%	100%	100% (5/5)
Prosthesis survival	Yes	Yes	Yes	100%
Functional loading maintained	Yes	Yes	Yes	All implants functional
Progressive crestal bone loss	None observed	None observed	Site-specific mesial angular change at #8	No generalized progressive pattern; #8 change noted
Peri-implant radiolucency	None	None	None	None
Implant mobility	None	None	None	None
Mechanical complications	None	None	None	None reported
Biologic complications	None	None	None	None reported
Esthetic outcome	Stable	Stable	Stable	Favorable soft tissue stability
Overall clinical outcome	Functional at follow-up	Functional at follow-up	Functional at follow-up	Descriptive long-term functional observation
Visible radiographic bone gain/fill	Not quantifiable from non-standardized images	Not quantifiable from non-standardized images	Not quantifiable; mesial angular change at #8 noted	No quantitative claim made
Final probing depths	2–3 mm	2–3 mm	2–3 mm	2–3 mm at all implant sites
Bleeding on probing	Within normal limits	Within normal limits	Within normal limits	No abnormal bleeding response reported
Percussion findings	No abnormal findings	No abnormal findings	No abnormal findings	Within normal limits

Because standardized calibrated radiographs were not obtained at treatment and follow-up, the available images do not permit reliable numerical measurement of marginal bone change. The radiographic outcome should therefore be interpreted as qualitative evidence of crestal bone maintenance rather than as a quantitative estimate of bone loss.

## Discussion

These cases illustrate an important point in long-term implant evaluation: survival is only part of the outcome. In the anterior maxilla, maintaining crestal bone and the overlying soft tissue architecture is important because changes in supporting bone can affect the restorative result. In this series, all five implants remained functional after 8-10 years, and the available serial radiographs generally showed a maintained crestal bone. The site-specific mesial angular crestal change at implant #8 in Case 3 is noted separately. These descriptive observations cannot establish causation or implant-specific efficacy.

Primary stability was also consistent across the three clinical situations, with insertion torque values between 35 and 45 Ncm. This is relevant because each case involved immediate placement and most sites required some form of regenerative treatment. Implant macrogeometry, thread design, surgical preparation, and local bone quality are recognized determinants of initial implant stability [2-5].

The tapered body and spiral thread design of the Ritter RI-Quadro Spiral implant are intended to increase bone engagement during insertion. Implant macrogeometry and thread configuration can influence insertion torque and primary stability, particularly in lower-density bone and post-extraction situations [2-4]. These principles provide a biologically and mechanically plausible explanation for the insertion torque achieved in the present cases, although this series was not designed to compare implant macrogeometries.

Crestal bone maintenance cannot be explained by insertion torque alone. Cervical microthreads have been proposed as a means of modifying stress distribution near the implant neck, but their clinical effect should not be overstated. A multicenter retrospective study of 1,780 platform-switched internal-connection implants found no statistically significant difference in marginal bone loss or implant survival between implants with and without microthreads [13].

The implant-abutment interface is another part of the equation. A systematic review reported that conical connections generally showed favorable abutment stability and seal performance and, in most included in vivo studies, less marginal bone loss than nonconical connections [7]. Platform switching has also been associated with less marginal bone loss than platform matching in systematic-review evidence [8]. Clinical evidence further indicates that marginal bone behavior around platform-switched conical-connection implants may be influenced by the vertical position of the implant relative to the crestal bone [14].

Surface topography also contributes to the bone response during healing. Systematic-review evidence indicates that titanium surface topography influences bone integration and that moderately rough surfaces generally produce a stronger bone response than smooth or minimally rough surfaces [1]. The relationship between implant surface characteristics and osseointegration is also supported by subsequent review literature [6].

The long-term findings should not, however, be attributed to the implant alone. Each case was managed with prosthetically driven positioning, atraumatic extraction, careful osteotomy preparation, and site-specific hard- and soft-tissue augmentation. Guided bone regeneration, connective tissue grafting, platelet-rich fibrin, and partial extraction therapy were used according to the clinical situation. In addition, restorative variables such as abutment height, transmucosal design, and prosthetic dimensions can influence marginal bone behavior [9-12].

This point is particularly relevant because all of the implants were placed in the esthetic zone and several sites began with compromised facial bone. The fact that the peri-implant architecture remained clinically and radiographically stable over many years is encouraging, but the outcome represents the combined effect of implant design and comprehensive treatment rather than evidence for any single design feature.

Patient demographics, smoking status, systemic health, and baseline periodontal status were available and are now reported for each case. Final follow-up probing depths, bleeding on probing, and percussion findings were also available and are reported. However, standardized maintenance intervals and other long-term variables, including plaque scores, keratinized tissue width, mucosal thickness, standardized esthetic indices, and patient-reported outcomes, were not collected uniformly, limiting assessment of potential confounding factors.

The principal limitations are the small number of patients, retrospective design, and lack of standardized calibrated radiographs. The original treatments were not planned as a prospective investigation, so mesial and distal bone changes cannot be reported in millimeters. Important long-term variables such as probing depth, bleeding on probing, plaque scores, keratinized tissue width, mucosal thickness, standardized esthetic indices, and patient-reported outcomes were also not collected in a uniform manner.

Future prospective studies should include a larger cohort, standardized parallel periapical radiographs with digital calibration, defined peri-implant clinical parameters, and statistical analysis of bone-level changes and complications. Comparative studies against other contemporary tapered implants would be useful in determining whether the favorable long-term pattern seen here is reproducible and whether specific implant design features provide a measurable clinical advantage.

## Conclusions

In this three-patient case series, five Ritter RI-Quadro Spiral implants placed in the anterior maxilla remained functional after approximately 8-10 years. The available serial periapical radiographs generally demonstrated maintenance of peri-implant crestal bone, with a site-specific mesial angular crestal change noted at implant #8 in Case 3. At final follow-up, peri-implant probing depths were 2-3 mm, bleeding on probing was within normal limits, and percussion testing revealed no abnormal findings.

These observations show that the five implants remained functional within the comprehensive, prosthetically driven treatment provided in these three cases. The findings are descriptive and hypothesis-generating and do not demonstrate implant-specific efficacy. They should not be interpreted as proof that implant design alone produced the long-term result. Larger prospective studies using standardized radiographic measurements and defined peri-implant clinical parameters are needed to confirm the degree of marginal bone stability and determine the relative contribution of implant geometry, surface characteristics, restorative connection, regenerative procedures, and maintenance.
